# Heartfelt Breakthroughs: Elevating Quality of Life with Cutting-Edge Advances in Heart Failure Treatment

**DOI:** 10.3390/jcdd11010015

**Published:** 2024-01-05

**Authors:** Ramprakash Devadoss, Gagandeep Dhillon, Pranjal Sharma, Ram Kishun Verma, Ripudaman Munjal, Rahul Kashyap

**Affiliations:** 1 Carle Methodist Hospital, Peoria, IL 61636, USA; ramprakash.devadoss@carle.com; 2 Department of Medicine, UM Baltimore Washington Medical Center, Glen Burnie, MD 21061, USA; gagandeep.dhillon@umm.edu; 3 Department of Medicine, Northeast Ohio Medical University, Rootstown, OH 44272, USA; sharma.pranjalmd@gmail.com; 4 Parkview Health System, Fort Wayne, IN 46845, USA; vermark2006@gmail.com; 5 Kaiser Permanente Medical Center, Modesto, CA 95356, USA; ripudaman.munjal@gmail.com; 6 WellSpan Health, 1001 S George St., York, PA 17403, USA

**Keywords:** heart failure, ARNI, SGLT2 inhibitors, gene therapies, artificial intelligence

## Abstract

Heart failure is a cardiovascular condition, leading to fatigue, breathlessness, and fluid retention. It affects around 56 million people globally and is a leading cause of hospitalization and mortality. Its prevalence is rising due to aging populations and lifestyle factors. Managing heart failure demands a multidisciplinary approach, encompassing medications, lifestyle modifications, and often medical devices or surgeries. The treatment burden is substantial, impacting patients’ daily lives and straining healthcare systems. Improving early detection, novel therapies, and patient education are crucial for alleviating the burden and enhancing the quality of life. There are notable advancements in the field of heart failure treatment and prevention. We will discuss significant pharmacological and device advances related to heart failure, including angiotensin receptor–neprilysin inhibitor, sodium–glucose co-transporter inhibition, glucagon-like peptide-1 agonist, cardiac resynchronization therapy, cardiac contractility modulation, mechanical circulatory support devices, and transcatheter valve interventions. We will also review novel therapies on the horizon, emerging technologies like CRISPR-based treatments for genetic anomalies, and the involvement of artificial intelligence in heart failure detection and management.

## 1. Introduction

**Figure 1:**
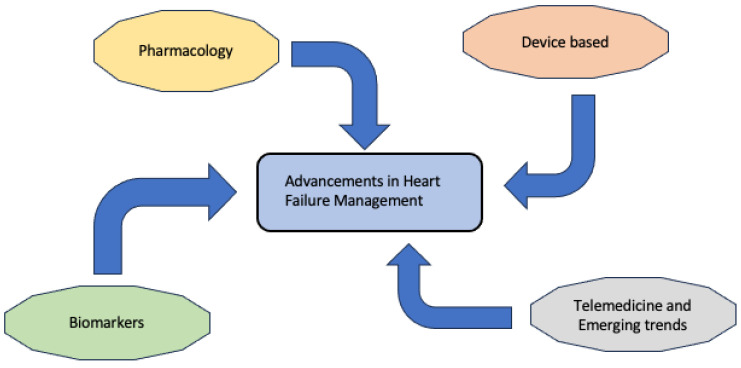
Central illustration.

Heart failure is caused by left ventricular dysfunction, resulting in clinical symptoms such as the shortness of breath, tiredness, and the limitation of exercise capacity. It is a major public health concern with an estimated prevalence of over 56 million globally, with an age-standardized rate (ASR) of 711.90 per 100,000 population [1]. Though there was an improvement in ASR from 1990 to 2019, ASR prevalence has increased at an annual percentage change of 0.6% from 2017 to 2019 [1]. A nationwide survey from the American Heart Association in 2013 reported that direct and indirect costs attributed to HF will significantly increase from USD 30.7 billion in 2012 to USD 69.7 billion in 2030 [2]. In attempts to reduce the morbidity and mortality of heart failure patients, multiple new therapeutic interventions have surfaced in the last decade. This review will focus on recent advancements significantly impacting heart failure management. We will discuss the topic under the following categories: pharmacological therapies, device-based interventions, biomarkers in heart failure, telemedicine, and emerging trends (Figure 1).

## 2. Classification

Heart failure has been classified according to the left ventricular ejection fraction (LVEF) [3]:

1. Heart failure with preserved ejection fraction (HFpEF: LVEF ≥ 50%);
2. Heart failure with midrange ejection fraction (HFmrEF: LVEF 41–49%);
3. Heart failure with reduced ejection fraction (HFrEF: LVEF ≤ 40%).

Multiple evidence-based interventions have evolved for HFrEF, refining treatment for better outcomes. On the contrary, HFpEF management, unfortunately, has not had any significant progress because of a lack of clear benefits of the same intervention in this patient population in randomized controlled trials. This highlights the distinct difference in the underlying pathology for these categories, contributing to differential treatment responses.

American College of Cardiology (ACC)/American Heart Association (AHA) provided various stages of heart failure (Table 1) to identify high-risk subgroups and target early interventions to reduce morbidity and mortality [4].

### HFpEF vs. HFrEF

There is significant variation in treatment responses between disease phenotypes. As discussed in detail below, there is contrast in benefits of most pharmacotherapies between HFrEF and HFpEF. This is likely secondary to dissimilarity in pathogenesis despite similar clinical symptoms. Multisystem abnormalities are common in HFpEF patients [5]. Different mechanisms including arterial hypertension causing adverse LV remodeling and pro-inflammatory co-morbidities causing microvascular endothelial cell inflammation and resultant left ventricular remodeling are some of the proposed theories. Defining the various phenotypes and the identification and targeting of treatment are active research areas in this field [5]. H2FPEF score proposed to be helpful in diagnosing HFpEF in patients presenting with unexplained dyspnea [6]. The score is calculated based on six variables: age, BMI, number of antihypertensive medications, atrial fibrillation history, pulmonary hypertension, and filling pressures based on echo parameters. A value of >6 is considered diagnostic of HFpEF. A value of <2 rules out HFpEF and additional testing suggested for intermediate values.

Unlike HFrEF, HFpEF lacks evidence-based therapeutic targets. Apart from recent trials showing the benefits of sodium–glucose cotransporter 2 inhibitors and glucagon-like peptide-1 agonists as discussed below, HFpEF management has traditionally focused on symptomatic management with decongestion and treating underlying co-morbidities such as diabetes, obesity, HTN, ischemic heart disease, and arrhythmia. Also, it has focused on identifying pathologies with proven therapeutic strategies such as hypertrophic cardiomyopathy, amyloid cardiomyopathy, high-output heart failure, primary pulmonary HTN, constrictive pericarditis, and cardiac sarcoidosis. Revascularization has been shown to improve HFpEF outcomes in observational studies [5]. Exercise training has also shown to improve peak oxygen uptake, 6 min walk distance, and quality of life in randomized controlled trials [7]. Physical rehabilitation has been shown to be helpful even in frail patients after being hospitalization for heart failure [8].

## 3. Pharmacological Therapies

### 3.1. ARNI (Angiotensin Receptor–Neprilysin Inhibitor)

Beta-blockers, angiotensin-converting enzyme inhibitors (ACEi), and mineralocorticoid receptor antagonists (MRA) are traditionally considered the cornerstone in the management of patients with HFrEF based on multiple trials with class 1 recommendations in guidelines [9]. PARADIGM-HF was the first trial to show significant clinical benefit of this new medication (Sacubitril–Valsartan). When compared to Enalapril, ARNI reduced cardiovascular mortality (13.3% vs. 16.5%; hazard ratio (HR): 0.8) and reduced hospitalization for heart failure (12.8% vs. 15.6%; HR: 0.79) [10]. This trial studied stable heart failure patients with LVEF ≤ 40% and was prematurely terminated because of its overwhelming benefit. Later, PIONEER-HF investigators showed significant improvement in NT-proBNP (N-terminal Pro-B-type natriuretic peptide), indicating an improvement in heart failure in HFrEF patients hospitalized for acute decompensation [11].

The same drug did not show significant improvement in heart failure hospitalizations or death from cardiovascular causes among patients with heart failure and LVEF ≥ 45% in the PARAGON-HF trial [12]. However, there was a considerable improvement in NT-pro BNP in patients with LVEF > 40%, and a recent worsening heart failure event with ARNI compared to Valsartan in the PARAGLIDE-HF trial [13]. 

Given the substantial benefit of ARNI, especially in HFrEF, ARNI had class I recommendations in patients with HFrEF in the latest reiteration of heart failure management guidelines by the American College of Cardiology [14]. Also, it recommended switching stable heart failure patients from ACEi/ARB to ARNI. 

### 3.2. SGLT2 Inhibitors (Sodium–Glucose Cotransporter 2 Inhibitors)

SGLT2 inhibitors reduce glycemia, blood pressure, body weight, and albuminuria in people with diabetes mellitus. EMPA-REG investigators showed a substantial benefit of empagliflozin in reducing primary outcomes inclusive of death from a cardiovascular cause, nonfatal myocardial infarction, or nonfatal stroke. The composite adverse event happened in 37.4 per 1000 patient-years in the empagliflozin arm vs. 43.9 per 1000 patient-years in the control arm (HR—0.86). Empagliflozin also reduced death from any cause and heart failure hospitalizations [15].

Subsequently, the CANVAS group reported improved cardiovascular mortality and morbidity with Canagliflozin in 2017 [16]. The composite primary outcome, like the EMPA-REG trial, occurred in 26.9 participants per 1000 patient-years in the intervention group compared to 31.5 participants in the control arm (HR—0.86). There was also a clear signal for reduced heart failure hospitalization with 5.5 vs. 8.7 events in the intervention and control arm, respectively (HR: 0.67). High amputation rates raised safety concerns in patients with previously diagnosed peripheral arterial disease.

**Figure 2:**
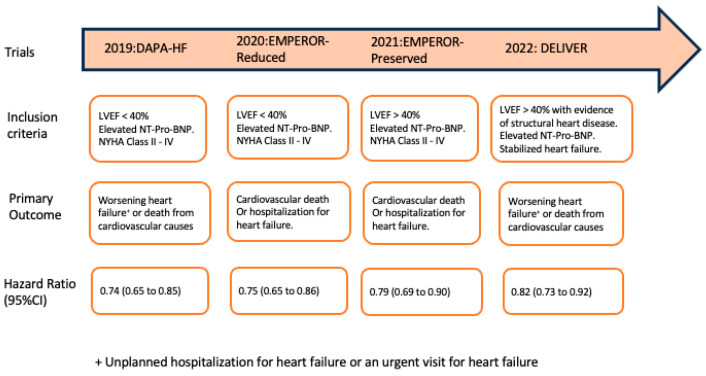
Clinical trials of SGLT2 inhibitors in stabilized heart failure patients irrespective of their diabetic status.

Later, the DAPA-HF and EMPEROR-Reduced trials were conducted specifically in HFrEF patients with or without diabetes. Both showed significant improvements in their primary outcome, which was heart failure hospitalization or cardiovascular death, with a hazard ratio of 0.74 with Dapagliflozin [17] and 0.75 with Empagliflozin [18] (Figure 2). These studies noted no significant safety concerns other than an increased risk of uncomplicated genital infections.

Then, the trial was repeated in other heart failure groups as well. Dapagliflozin [19] and Empagliflozin [20] reduced heart failure hospitalizations in patients with heart failure and LVEF > 40% (Figure 2). SGLT2 inhibitors are the only medication class that has shown benefit in patients with HFpEF in randomized controlled trials. SGLT2 inhibitors have the highest rank of recommendations for patients with HFrEF in the 2022 ACC/AHA guidelines [14].

### 3.3. Vericiguat

**Figure 3:**
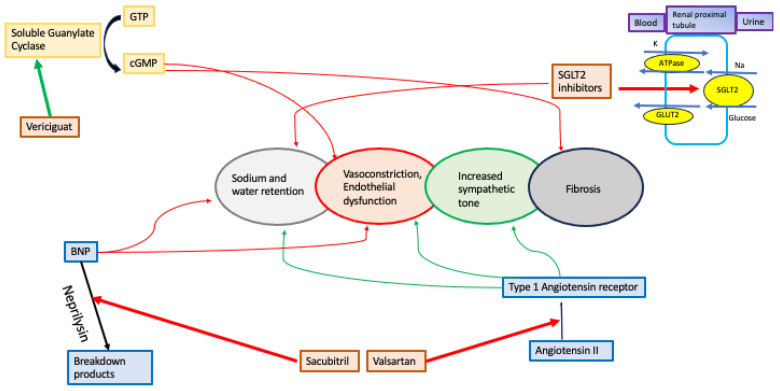
Key elements of heart failure and newer therapeutic targets [Red arrow: inhibition; Green arrow: Increase; Blue arrow: direction of movement; Black arrow: Conversion].

Modulating the nitric oxide-soluble guanylate cyclase pathway that generates cyclic GMP (guanosine 3′,5′-cyclic monophosphate) is essential for normal cardiovascular function. In heart failure, endothelial dysfunction and reactive oxygen species lower nitric oxide bioavailability, resulting in a relative deficiency of soluble guanylate cyclase and reduced cyclic GMP generation. Vericiguat enhances the cyclic GMP pathway by directly stimulating soluble guanylate cyclase through a binding site independent of nitric oxide (Figure 3).

This novel oral soluble guanylate cyclase stimulator showed a reduction in the primary outcome, a composite of death from cardiovascular causes and hospitalization for heart failure in patients with heart failure and LVEF < 45% with evidence of recent worsening of heart failure warranting hospitalization or outpatient intravenous diuretic therapy. Heart failure hospitalizations predominantly drove the positive response. The study patients had a worse NYHA class and higher NT-ProBNP levels than ARNI and SGLT2 trials, indicating a high-risk subset. The difference was notable after three months of treatment [21]. Given its vasodilating properties, Vericiguat resulted in symptomatic hypotension and syncope, although these were not significantly higher than placebo. The median follow-up time in the study was 10.8 months. A longer follow-up time may show more promising results in future studies. 

### 3.4. GLP (Glucagon-like Peptide)-1 Agonist

Multiple earlier trials evaluating GLP-1 receptor and cardiovascular outcomes did not focus on heart failure outcomes in their primary endpoints [22]. In the HARMONY trial, Albiglutide showed a reduction in heart failure hospitalization (HR—0.71). The trial did not include baseline LVEF or NYHA classification. STEP-HFpEF trial was the recent GLP-1 trial focusing on HFpEF, showing improvements in symptoms and exercise function [23]. GLP-1 Agnoist are the second class of drugs to offer some benefit in randomized controlled trials for patient with HFpEF. 

In another recent trial, Semaglutide was shown to have cardiovascular benefits in obese patients even in the absence of diabetes mellitus [24]. In this study, a reduction in the composite heart failure endpoint (cardiovascular death or hospitalization or an urgent medical visit for heart failure) was observed. The hazard ratio was 0.81, which was statistically significant. A significant discontinuation of Semaglutide (~16.6%) was noted in the trial because of its side effects. The predominant side effect was gastrointestinal disorders. Baseline NYHA classification or LVEF was not included in this study. 

## 4. Device-Based Interventions

### 4.1. Cardiac Resynchronization Therapy (CRT)

CRT is an advanced pacemaker option with an additional left ventricular pacing lead via coronary sinus in patients with conduction system disease, causing dyssynchrony to improve LVEF, and it was evaluated in 2005 in the CARE-HF [25] trial and then in the MADIT-CRT [26] trial in 2009. It is most beneficial for patients with HFrEF and Left Bundle Branch Block (LBBB) with QRS duration of more than 150 milliseconds. Patients with QRS complexes duration of 120–149 milliseconds or non-LBBB morphology and HFrEF may also benefit from this device depending on their baseline NYHA functional class and LVEF [14]. A recent BUDAPEST trial showed the significant benefits of upgrading from a dual-chamber pacemaker to CRT in patients with a high burden of RV pacing (≥20%), wide QRS complex (≥150 milliseconds), and reduced ejection fraction (≤35%) [27].

### 4.2. Transcatheter Mitral Valve Interventions

Mitraclip is a transcatheter intervention option for mitral valve regurgitation in patients at high risk for surgical interventions. COAPT (Cardiovascular Outcomes Assessment of the MitraClip Percutaneous Therapy for Heart Failure patients with Functional Mitral Regurgitation) trial favored the intervention in this group with a significant reduction in heart failure hospitalization (HR: 0.53) and all-cause mortality (HR: 0.62) [28]. However, MITRA-FR (Percutaneous Repair with Mitraclip Device for Severe Functional/Secondary Mitral Regurgitation) did not benefit significantly from this technology in a similar patient population in terms of mortality or heart failure hospitalization [29]. Multiple proposed theories exist [30] for the discrepant results. Overall, the evidence favors this intervention in appropriate patients. Transcatheter edge-to-edge has class II(a) recommendation for HFrEF and HFmrEF patients with NYHA (New York Heart Association) class II-IV, severe secondary mitral regurgitation, suitable anatomy, LV end-systolic dimension ≤ 70 mm, and PASP ≤ 70 mm Hg [14]. 

### 4.3. Mechanical Circulatory Support (MCS)

Multiple trials are ongoing in this space, assessing the role of MCS in managing heart failure, including temporary and permanent devices. Temporary trans-axial pumps, like Impella and Abiomed Inc., and extracorporeal pumps, like Tandem Heart and Cardiac Assist Inc., are being currently used in the percutaneous revascularization of complex coronary disease patients who are not candidates for surgical intervention and heart failure patients with cardiogenic shock refractory to pharmacological interventions. Trials so far have not shown any significant clinical benefit with the unselected use of temporary MCS in patients with cardiogenic shock complicating acute myocardial infarction [31]. These devices have a role in the acute decompensation of chronic heart failure patients as a bridge to decisions regarding long-term management options—Class II(a) recommendation in ACC/AHA heart failure management guidelines [14].

The Multicenter Study of MagLev Technology in Patients Undergoing Mechanical Circulatory Support Therapy with HeartMate 3 (MOMENTUM 3) trial showed significant benefit from a magnetically levitated centrifugal pump compared to traditional axial continuous-flow pumps for advanced heart failure patients, warranting implantable pumps for support. Reoperation for pump malfunction secondary to thrombosis was less frequent in the centrifugal pump group [32]. Durable left ventricular assist devices have the class I recommendation for HFrEF patients with NYHA class IV symptoms, despite optimal medical therapy, or those deemed dependent on IV inotropes as per heart failure guidelines [14].

### 4.4. Cardiac Contractility Modulation (CCM)

Cardiac contractility modulation (CCM), a device-based therapy that involves applying relatively high-voltage, long-duration electric signals to the RV septal wall during the absolute myocardial refractory period, has been associated with the augmentation of LV contractile performance. FIX-HF-5 trial showed CCM-improved exercise tolerance and quality of life in heart failure patients with LVEF ≥ 25% and ≤45%, QRS duration < 130 ms, and NYHA class III or IV symptoms, leading to its approval for use in the United States [33]. Long-term follow-up of patients with CCM on the CCM-REG registry showed improved functional status, quality of life, LVEF, and heart failure hospitalization rates [34].

### 4.5. Obstructive Sleep Apnea (OSA) and Heart Failure

Sleep apnea is prevalent yet underdiagnosed and untreated in cardiovascular patients. A recent retrospective study showed that cardiac patients with sleep apnea treated with continuous positive airway pressure (CPAP) were likely to have a 60% reduction in readmission in 30 days [35]. Untreated sleep apnea impacts cardiovascular health with increased sympathetic activity, oxidative stress, endothelial dysfunction, and metabolic dysregulation [36]. Since compliance with CPAP is still challenging, newer treatment options are available. These include hypoglossal nerve stimulator therapy, oral appliances, positional therapy, oral negative pressure devices, and eXciteOSA therapy.

## 5. Biomarkers in Heart Failure

Biomarkers in heart failure can be loosely arranged into the following categories: (1) myocardial stress/injury, (2) neurohormonal activation, (3) remodeling, and (4) co-morbidities. 

B-type natriuretic peptide (BNP) and its biologically inert, amino-terminal pro-peptide counterpart (NT-proBNP) are the most common biomarkers to diagnose and determine HF prognoses. The most potent BNP inducer is the stretch of the left ventricular wall caused by increased pressure or volume. BNP can induce diuresis and cause vasodilation, renin–aldosterone, and fibrosis inhibition. As the degradation of BNP by neutral endopeptidases such as neprilysin is inhibited by ARNI, it becomes challenging to interpret the values in this population. BNP levels on admission were shown to be associated with in-hospital mortality risk in the Acute Decompensated Heart Failure National Registry (ADHERE) registry [37]. Discharge BNP level was also helpful in predicting one-year mortality in the Organized Program to Initiate Lifesaving Treatment in Hospitalized Patients with Heart Failure (OPTIMIZE-HF) trial [38]. Biomarker-guided heart failure therapy using BNP has shown to be superior to the standard of care with reduced event rates, improved quality of life, and favorable effects on cardiac remodeling [39].

New biomarkers gaining importance in heart failure management include mid-regional pro adrenomedullin (MR-proADM) and copeptin (stable C-terminal pro-peptide fragment of arginine vasopressin), indicating neurohormonal activation, ST2, and Galectin-3, indicating myocardial remodeling. 

Adrenomedullin (ADM) is a vasodilatory peptide expressed in different tissues with potent hypotensive effects, and its levels are known to be elevated in patients with chronic heart failure. Because of its biologically stable nature, immunoassays targeting prohormone fragments such as MR-pro ADM and mid-regional pro-atrial natriuretic peptide (MR-ProANP) as surrogate markers were developed. In a prospective multicenter trial, MR-proADM was shown to have independent prognostic value, predicting the 90-day mortality risk and adding prognostic value to BNP [40].

Arginine vasopressin (AVP) is a posterior pituitary hormone known to have antidiuretic and vasoconstrictive properties. Its concentrations are elevated in heart failure patients and postulated to mediate hyponatremia, which is a poor prognostic marker. Co-peptin is the C-terminal segment of preprovasopressin identified to be a stable and reliable surrogate marker. In the same multicenter study, elevated co-peptin predicted increased 90-day mortality, readmissions, and emergency department visits [41].

Galectin-3, a member of the galectin family, was shown to be of both diagnostic and prognostic value in heart failure patients [42]. Galectin-3 has been postulated to be involved in cardiovascular remodeling and used as a biomarker for fibrosis and inflammation. Studies have also shown ethnic difference in the Galectin-3 prognostic value with limited utility in the African American population based on the limited observation data [43]. Galectin-3 values have been shown to be predictive of survival post left ventricular assist device placement and also coronary allograft vasculopathy post-transplant in a prospective study [44].

ST2 is strongly induced in the setting of cardiomyocyte or cardiac fibroblast stretch. ST2 is closely involved in LV hypertrophy, fibrosis, and remodeling due to its interaction with IL-33. Increasing ST2 concentrations are associated with adverse clinical outcomes in HF and are unaffected by BMI or renal function. Elevated ST2 levels have also been shown to predict the development of heart failure in at-risk populations like patients with acute MI [45], providing an opportunity for early identification and targeting treatment. Large prospective trials are needed to assess these new markers in biomarker-guided heart failure management.

## 6. Telemedicine and Emerging Trends in Heart Failure Management

### 6.1. Remote Pulmonary Pressure Monitoring

CardioMems, an implantable pressure sensor placed in the pulmonary artery, reduced heart failure-related hospitalizations due to its ability to track patients’ filling pressures and to guide management [46]. The study included all heart failure patients in NYHA class III, irrespective of the left ventricular ejection fraction and a previous hospital admission for heart failure. The benefit was again reproducible in the GUIDE-HF trial, reducing heart failure hospitalizations across the spectrum of LVEF, but was more prominent in the HFrEF subgroup [47]. Post-FDA approval, the real-world observational study also showed significantly lower heart failure and all-cause hospitalization post-device placement [48]. This technology greatly benefits heart failure patients with its ability to monitor and intervene remotely to prevent exacerbation, warranting hospitalization.

### 6.2. Telerehabilitation

Exercise-based interventions have consistently demonstrated a significant, clinically meaningful improvement in symptoms, objectively determining exercise capacity and quality of life in heart failure patients [49]. Telerehabilitation is a home-based program with devices to monitor vitals and an online platform for structured exercise regimens. This field is still in its infancy, but with the advent of new technology, it is evolving rapidly. Telerehabilitation will improve access to many patients with rehabilitative needs with travel limitations.

### 6.3. Artificial Intelligence in Heart Failure

Artificial intelligence (AI) has seen a rapid increase in utility in medicine in recent years. AI is increasingly used to revolutionize risk assessment, screening, diagnosis, treatment and drug discovery in cardiovascular medicine [50]. EAGLE trial is testing AI-guided ECG screening for low ejection fraction, which will significantly impact the heart failure field with the early identification of at-risk populations [51]. Multiple supervised and semi-supervised machine learning (ML) algorithms have predicted the onset of heart failure based on large labeled and unlabeled datasets from electronic health records. However, it is important to compare the performance of these ML-developed risk models to known or conventional approaches to determine their clinical utility [52]. Some ML algorithms also incorporate imaging data, which helps to track change longitudinally and predict disease progression. 

Heart Failure Association (HFA) of the European Society of Cardiology (ESC) recently provided guidelines for developing HFpEF models through a stepwise approach of comprehensive cardiac and extra-cardiac phenotyping. There were three leading phenogroups based on aging, cardiometabolic stress, and chronic hypertension [53]. ML algorithms can be beneficial in identifying phenotypes and provide a hypothesis-generating framework for designing future clinical trials. 

AI models have been used to assess heterogeneity in response to HF pharmacotherapies [54] and cardiac resynchronization therapy (CRT). At least six trials have studied machine learning algorithms for predicting response to CRT [52]. Studies have identified several predictors like sex, etiology, severity of HF, renal function, and comorbidity burden for determining the response to CRT. ML analytics demonstrated predicting rehospitalization for heart failure with 87.5% sensitivity and 85% specificity based on non-invasive remote monitoring in the LINK-HF trial [55].

The incorporation of AI technologies into heart failure also faces several regulatory concerns. New privacy and data management principles are necessary that can allow for the training of algorithms in these datasets while also maintaining individual privacy [52]. The algorithms should undergo rigorous testing and validation to ensure proper performance. The US FDA has issued guidance emphasizing the prospective validation of AI algorithms before their implementation in clinical practice [56].

### 6.4. Gene Therapies for Advanced Heart Failure

There is a dysregulation of the excitation–contraction coupling at multiple levels in HF. Targets for gene therapy so far have involved various ways to restore contractile function, angiogenesis, cytoprotection, and stem cell homing.

The key regulator in cardiac contractility is the β-adrenergic system. It is downregulated and desensitized in HF because the critical protein G protein-coupled receptor kinase 2 (GRK2) is upregulated. In rodents and preclinical large animal heart failure models, the inhibition of GRK2 via βARKct (carboxyl-terminus of the β-adrenergic receptor kinase) expression has shown positive results including an improvement of the left ventricular systolic dysfunction [57]. Ca^2+^-handling proteins involved in the excitation–contraction coupling has also been assessed as targets for heart failure management. SERCA_2a_ gene transfer improved cardiac contractility in the swine volume-overload model of HF [58] and decreased arrhythmias and mortality [59]. Other studies have demonstrated the increase in the small ubiquitin-like modifier type 1 (SUMO1) with the help of the adenovirus vector leads to the increased levels of the SERCA2a gene, which results in improved cardiac contractility, decreased arrhythmias, and decreased mortality [60]. 

However, the same results as in animal models have been hard to reproduce in human trials. The initial Calcium Upregulation by Percutaneous Administration of Gene Therapy in Cardiac Disease (CUPID) [61] trial did demonstrate some benefits. However, the larger CUPID2 trial failed to demonstrate any significant benefit in the recurrence of heart failure or mortality [62]. AGENT-HF trial [63] showed similar results. Adenylyl Cyclase VI (AC VI) is another target that has been studied [64] and is awaiting phase III study [65]. Learning from the trials conducted so far, there is a focus for identifying new targets and improving the vector with high cardiac tropism that de-targets the liver [66].

Gene-editing technology has also evolved in recent years, leading to fundamental upgrades of the biomedical research model with the achievement of falling off-target incidence, improving editing efficiency, and expanding application scope. Current third-generation gene-editing technology clustered regularly interspaced short palindromic repeats (CRISPR)/CRISPR-associated protein 9 (Cas9) system functions through protein–nucleic acid complex [67]. A milestone advancement in genetic therapy leading to an effective and sustained improvement in patients with heart failure can be anticipated soon.

## 7. Conclusions

Despite medical advances, heart failure remains a major health issue, resulting in significant morbidity and healthcare expenses. Continued efforts in refining medical therapy targeting these high-risk populations have recently shown promising results. With few evidence-based therapeutic options, heart failure with preserved ejection fraction remains a challenge. Using artificial intelligence to identify at-risk groups and instituting early, appropriate risk factor modifications might be the key for reducing the global disease burden. Gene therapy with advanced gene-editing technology will be the next major milestone in managing heart failure.

## Figures and Tables

**Table 1:** ACC/AHA heart failure stages.

Stage A	Patients at risk for HF but have no symptoms or structural heart disease.
Stage B	Patients have structural heart disease but are asymptomatic.
Stage C	Patients have structural heart disease plus symptoms.
Stage D	Patients have refractory HF that requires modified interventions.

## Data Availability

No new data were created in this study.
